# Diagnostic Accuracy of Cytology Smear and Frozen Section in Glioma

**DOI:** 10.31557/APJCP.2019.20.2.321

**Published:** 2019

**Authors:** Anani Aila Mat Zin, Sarah Zulkarnain

**Affiliations:** *Department of Pathology, School of Medical Science, Health Campus, University Sains Malaysia, Kelantan, Malaysia. *

**Keywords:** Glioma, cytology smear, frozen section, diagnostic accuracy

## Abstract

Glioma is the commonest primary intracranial tumour and it has been the most predominant tumour in many studies. It accounts for 24.7% of all primary brain tumour and 74.6% of malignant brain tumour. Intraoperative diagnosis plays a crucial role in determining the patient management. Frozen section has been the established technique in providing rapid and accurate intraoperative diagnosis. However due to some disadvantages like ice crystal artefact, high expenditure and requirement of skilled technician, there is increase usage of cytology smear either replacing or supplementing frozen section technique. The aim of this review is to determine the diagnostic accuracy of cytology smear and frozen section in glioma and to see whether there is significant difference between those techniques. The overall diagnostic accuracy for frozen section in glioma ranging from 78.4% to 95% while for cytology smear, the diagnostic accuracy ranging from 50% to 100%. Based on certain literatures, no statistically difference was observed in diagnostic accuracy of cytology smear and frozen section. Thus, cytology smear provides an alternative method in establishing intraoperative diagnosis. Both cytology smear and frozen section are complimentary to each other. It is recommended to use both techniques to improve the diagnostic accuracy in addition with adequate knowledge, clinical history, neuroimaging and intraoperative findings.

## Introduction

Glioma is a central nervous system tumour arises in glial tissue and it can be astrocytoma, oligodendroglioma, ependymoma or mixture of these tumours.Worldwide, glioma is the most common primary intracranial tumour. It accounts 24.7% of all primary brain tumour and 74.6% of malignant brain tumour (Gupta et al., 2005). It also has been the predominant neoplastic lesion in most of the studies (Pawar et al., 2009; Rao et al., 2009; Mitra et al., 2010; Shrestha et al., 2014; Chand et al., 2016; Amraei et al., 2017; Cheunsuchon et al., 2017). Intraoperative histopathological diagnosis plays a crucial role in evaluating the sample adequacy, determining the diagnosis and tumour margins, optimizing the surgical procedure, post-surgical follow up and treatment. In certain situation, it can detect an unexpected lesion that cannot be determined by clinical or radiological imaging. This will reduce the surgical related morbidity and avoid the need for unnecessary second invasive procedure. Although advances of neuroimaging have been established, histopathological examination remains the gold standard in diagnosing glioma (Brat et al., 2008). Frozen section has been the established intraoperative histopathological evaluation worldwide since it was first introduced in 1891. Subsequently, cytology smear was introduced later and there was an increased usage of this technique for supplementing or replacing the use of frozen section technique due to advent of stereotactic procedures and the need for rapid intraoperative consultation (Jaiswal et al., 2012; Nanarng et al., 2015). Cytology smear technique includes crush preparation and touch imprint. In crush preparation, the fresh tissue is gently crushed on a glass slide by a second slide held at right angle. Meanwhile, the fresh tissue is gently touched on a glass slide using a forcep in touch imprint.

Both air dried smears and 95% ethyl alcohol fixed imprints will be stained using haematoxylin and eosin stains. Both cytology and frozen are important diagnostic tools in providing rapid and accurate intraoperative diagnosis. However in some hospital centres, frozen section facilities are limited and only cytology smear is available. The latter technique can only be applied in diagnosing intraoperative glioma replacing frozen section if there is no difference in diagnostic accuracy. Thus, the rationale of this article is to determine the diagnostic accuracy of cytology smear and frozen section in glioma and to see whether there is significant difference between those techniques.

**Table 1:** Diagnostic Accuracy of Frozen Section

Author(s)/ Year	Period of study (year)	No of cases	Result(S)
(Samal et al., 2017)	2.5	63	The complete correlation/diagnostic accuracy of cytology for glial tumours are 87.5%. The sensitivity, specificity, positive predictive value in detecting neoplastic condition were 96.15%, 75%, 96.15% and 75%.
(Tofte et al., 2014)	4	578	Diagnostic accuracy for low grade glioma was 78.4% where 13.5% was upgraded to high grade glioma. Sensitivity for low grade glioma was 64.4% while specificity was 96.7% with positive predictive value of 78.4% while negative predictive value of 93.7%. In high grade glioma, the diagnostic accuracy was 91.6% where 4.2% was downgraded to low grade glioma. The sensitivity was 79.1%, specificity was 95.5% with positive predictive value of 91.6% while negative predictive value of 88.1%. The overall diagnostic accuracy was 90.3%
(Mitra et al., 2010)	1.5	114	The diagnostic accuracy for glioma was 86.8%. The overall diagnostic accuracy was 90.6%. 4 cases were under graded.
(Mukherjee et al.,2015)	1	55	The diagnostic accuracy in glial tumours was 96.06%
(Chand et al., 2016)	1.5	90	The overall diagnostic accuracy was 95%; with sensitivity and specificity of 96.8 % and 100 %, respectively.

**Table 2:** Diagnostic Accuracy of Cytology Smear

Author	Period of study (years)	No of cases (n)	Conclusion
(Samal et al., 2017)	2.5	63	The complete correlation/diagnostic accuracy of cytology for glial tumours are 88.24%. The sensitivity, specificity, positive predictive value in detecting neoplastic condition were 94.4%, 85.7%, 98.07% and 66.67%.
(Sharma and Deb, 2011)	1	90	Pilocytic astrocytoma, ganglioglioma, glioblastoma multiforme and ependymoma achieved 100 % diagnostic accuracy. 71.4% of accuracy was observed in low grade glioma as one of the discordant was upgrade to anaplastic astrocytoma on histopathological examination. Only 50 % of accuracy in high grade glioma as 1 out of 2 cases was misdiagnosed as high grade glioma which final result was metastatic tumour
(Kini et al., 2008)	3	100	Astrocytoma grade I, II, III and ependymoma achieved 100% diagnostic accuracy whereas only 64.7% and 22.2% were observed in glioblastoma and oligodendroglioma & mixed glioma, respectively. Mixed glioma was missed due to predominance of one histological type. Anaplastic astrocytoma was misdiagnosed due to lack of nuclei uniformity and variable cytoplasmic processes. The overall diagnostic accuracy was 86%
(Patil et al., 2016)	-	50	87.50% diagnostic accuracy in astrocytic tumour. 2 cases of glioblastoma were under diagnosed as anaplastic astrocytoma due to sampling error where characteristic necrosis was absent. The overall diagnostic accuracy was 92%
(Mitra et al., 2010)	1.5	114	The diagnostic accuracy for glioma was 84.9%. The overall diagnostic accuracy was 88.5%
(Chand et al., 2016)	1.5	90	The overall diagnostic accuracy was 91.25% with a sensitivity and specificity of 95.5% and 100% respectively.

**Table 3:** The Cytomorphology Discrepancy Seen in Cytology Smears for the Common Glial Tumours are Summarized as Follows

Author	Cytomorphology	Tumour grade
(Savargaonkar and Farmer, 2001; Liu et al., 2002; Pawar et al., 2009, Nanarng et al., 2015)	Moderate cellularity smear with mild nuclear pleomorphism and minimal anisocytosis in the fibrillary background. The chromatin is fine to coarse granular. The cytoplasm is scanty and show variable processes. No mitosis, necrosis or endothelial proliferation is observed.	Low grade astrocytoma
(Sharma and Deb, 2011; Shrestha et al., 2014)	Biphasic pattern composed of marked elongated bipolar cells displaying bland spindle nuclei with microcystic area. Many Rosenthal fibers and eosinophillic granular bodies are appreciated.	Pilocytic astrocytoma
(Sharma and Deb, 2011)	Hyperchromatic nuclei with irregular nuclear outline, coarse chromatin. Presence or absent of gemistocytic phenotype embedded in the meshwork of glial fibres.	Diffuse astrocytoma
(Kini et al., 2008; Nanarng et al., 2015)	Increase cellularity with closely packed cell and distinct papillary pattern. The nuclei are hyperchromatic and pleomorphic with coarse chromatin and prominent nucleoli. There are increase in mitotic figures and vascular proliferation.	Anaplastic astrocytoma
(Kini et al., 2008; Nanarng et al., 2015)	High cellularity smear with discohesive cells. There is marked nuclear pleomorphism, atypia and multinucleation. Significant mitotic figures and necrosis are observed along with marked endothelial proliferation and glomeruloid bodies.	Glioblastoma
(Pawar et al., 2009; Sharma and Deb, 2011; Shrestha et al., 2014; Nanarng et al., 2015)	Moderate to high cellularity smears composed of discohesive uniform, relatively small round cells, well-defined cell margins with mildly pleomorphic dark nuclei and inconspicuous nucleoli. The cytoplasm is scanty and clear. The fine granular background is lack of fibrillary material. Prominent thin walled vessel and foci of calcification are seen in certain cases.	Oligodendroglioma
(Pawar et al., 2009; Sharma and Deb, 2011)	In low grade ependymoma, the tumour composed of monomorphic population of round cells with salt-and pepper-like chromatin and conspicuous micronucleoli with scanty cytoplasm in fibrillary background. The cells are arranged in papillary pattern. Prominent perivascular pseudorosette is the main characteristic in this type of tumour.	Ependymoma

From the Table 1 and Table 2, we can conclude that the diagnostic accuracy for frozen section in glioma ranging from 78.4% to 95% while for cytology smear, the diagnostic accuracy was ranging from 50% to 100%.

According to (Sharifabadi et al., 2016; Samal et al., 2017), they concluded that there was no statistically difference in diagnostic accuracy of crush preparation and frozen section in intraoperative diagnosis. The p value was > 0.05. Thus, cytology smear can be used as a replacement or supplementing technique to frozen section. This is basically important when frozen section facilities and skills are unavailable, unequivocal result from frozen section technique or tissue sample is limited. 

In some studies, the accuracy of intraoperative diagnosis increases when both techniques are used (Brommeland et al., 2003; Rao et al., 2009; Sharifabadi et al., 2016). It is said that both techniques complemented each other in getting the accurate diagnosis.

*Frozen section or cytology smear*

A rapid and accurate intraoperative diagnosis is mandatory in determining the patient management. Frozen section has been the established technique as it provides good architectural detail of the lesion and display better histologic pattern and cytomorphology (Sharma et al., 2011). For example, palisading necrosis and microvascular proliferation are better appreciated in frozen section in a case of high grade glioma (Cheunsuchon et al., 2017). This technique is said to be useful in diagnosing firm and rubbery central nervous system tumour such as meningioma, ependymoma and metastatic tumour (Al-Ajmi et al., 2016). In glioma, frozen section showed poor quality slide preparation due to its soft nature (Nanarng et al., 2015). 

 The diagnostic accuracy of frozen section for glioma is limited. This is due to ice crystal artefact causing distortion of architectural details which can lead to over grading of glioma (Plesec and Prayson, 2007; Nanarng et al., 2015; Patil et al., 2016). Furthermore, trained staff and expensive equipment are also needed to prepare the slide where in some countries these facilities are lack.

Meanwhile, the cytology smear is simple, rapid, technically easier and reliable. It requires only a small piece of tissue for each slide especially when the tissue is limited and further paraffin section is needed (Sharifabadi et al., 2016). This technique is significantly useful in soft and friable tumour such as in glioma as it can show good cellularity on smear (Nanarng et al., 2015; Patil et al., 2016). In addition, the nuclear and cytoplasmic details are better observed by cytology smear (Kumar et al., 2013). 

Based on literatures, squash smear is superior to frozen section in demonstrating the characteristic features of glioma such as thick and coarse glial fibril background, rosettes, pseudo-rosettes and Rosenthal fibres (Sharifabadi et al., 2016; Cheunsuchon et al., 2017). The main disadvantages of squash smear are crush or overstretch artefact and difficulty in interpreting the slide if the smears are too thick (Rao et al., 2009).

Meanwhile, touch imprint is best for cellular lesion and demonstrate better morphological details without crushing artefact that are encounter in squash smear preparation (Sharifabadi et al., 2016). The major disadvantage of this touch imprint technique is poor cellular yield which reduce the diagnostic accuracy of this technique (Nanarng, Jacob et al., 2015). Careful handling is also needed in the preparation of touch imprint slide as the architecture might be distorted due to soft nature of the specimen (Krishnappa et al., 2017).

*Cytomorphology*

Based on literatures, it is unwise and careful caution should be practise upon categorizing and grading glioma in rapid intraoperative diagnosis. This is mainly due to heterogeneity of the tumour making it difficult to categorize and grade glioma (Pawar et al., 2009; Mitra et al., 2010). It is reported that glioma had attributed the highest discrepancy of cases (Cheunsuchon et al., 2017). One of the common reasons causing discordant of result is sampling error (Ud Din et al., 2011; Khamechian et al., 2012; Kumar et al., 2013). It includes inadequate sample or sampling at the wrong site.Due to this reason, making an intraoperative diagnosis can be quite difficult and there are higher chances of making an error. This especially important in mixed gliomas where difficulties are encounter due to predominance of one histological subtype. The other component may be missed especially if they are lesser in quantity and bland with dense fibrillary background of the astrocytic components (Kini et al., 2008). Other than that, high cellularity and nuclear pleomorphism might be wrongly interpreted especially in thick smear (Nanarng et al., 2015). This eventually causing overgrading of the tumour. Interference like necrosis and inflammation might also obscure the true morphology of the tumour (Pawar et al., 2009). Due to this reason, the surgeon will try to avoid sampling from necrotic area. Unfortunately, this action will sometimes attributed to tumour undergrading (Mitra et al., 2010). In a case of oligodendroglioma, freezing tissue often alter the morphology of the nucleus causing nuclear irregularity. This will make the tumour cells look similar to astrocytoma (Krishnappa et al., 2017). Other than that, a prompt fixation is needed to visualize perinuclear halo in oligodendroglioma.

*Development of intraoperative molecular genotyping test in glioma*

Challenges might happen in the situation of low grade glioma (grade II and III glioma), low cellularity yield and small stereotactic biopsy specimen. Fortunately, recent studies have identified somatic mutation in the isocitrate dehydrogenase 1 and 2 (IDH 1 and 2) as well as Telomerase Reverse Transcriptase (TERT) promoter genes which present over 80% of glioma (Kurimoto et al., 2016). This has led to development of molecular genotyping test to detect these types of gene mutation using polymerase chain reaction (PCR) method.It required about 60-65 minutes to obtain the result (Shankar et al., 2015; Kanamori et al., 2014). Despite longer period of time required in comparison with frozen and cytology smear, it has good sensitivity and specificity for WHO grade II and III glioma with 96% sensitivity and 100% specificity (Shankar et al., 2015). Other than that, it may help in differentiating low grade glioma with other tumour or reactive gliosis (Kurimoto et al., 2016). As a result, maximum tumour removal is achieved and repeated operative procedure can be avoided. Other test such as mass spectrometry and immunohistochemistry test are also available in detection of IDH1 mutation (Santagata et al., 2014; Wirsching and Weller, 2016).

In conclusion, frozen section technique is rapid and able to demonstrate the architectural characteristic however the major drawback of using this technique is freezing artefact. Besides that, this technique requires trained staff and expensive equipment. Based on many literatures, the diagnostic accuracy of cytological smear is almost equal to that of frozen section technique. Furthermore, cytology smear technique (squash smears and touch imprints) is rapid, less expensive, and technically much easier and requires only a small piece of tissue for each slide. It provides an alternative method when facilities for frozen section and skills are unavailable. Many studies have been proved that cytology smear and frozen section are complimentary to each other. It is recommended to use both techniques to improve the diagnostic accuracy if facilities and skills are available. Other than that, a trained neuropathology in addition with good clinical history, neuroimaging and intraoperative findings are crucial in yielding higher diagnostic accuracy. 

## Authorship contributions

Study concepts/ review-analysis/ manuscript drafting and approved the final version: all authors

## Conflict of interests

None.
